# Hypoglycaemic unawareness: A systematic review of qualitative studies of significant others' (SO) supportive interventions for patients with diabetes mellitus

**DOI:** 10.1016/j.heliyon.2018.e00887

**Published:** 2018-11-02

**Authors:** E. Hartill, R.B. Gillis, S. Imran Jiwani, N. Recchia, A. Meal, G.G. Adams

**Affiliations:** The University of Nottingham, Faculty of Medicine and Health Sciences, C Floor, South Block Link, Queen's Medical Centre, Nottingham, NG7 2HA, UK

**Keywords:** Medicine, Health profession, Public health

## Abstract

**Background:**

Hypoglycemia unawareness (HU) has been attributed to both a downward shift in central nervous system (CNS)-triggered sympatho-adrenal responses to low glycaemic thresholds and a subsequent loss of adrenergic symptoms, which, in addition, to cerebral cortex adaptations permit normal function under hypoglycaemic conditions. Both of these mechanisms are brought about by recurring hypoglycemic events (hypoglycemia-associate autonomic failure, HAAF). This can contribute to repetitive cycles of increasingly severe hypoglycaemia, the consequences of which have considerable impact on relatives and significant others (SO) when providing care to patients with diabetes.

**Methods:**

A Systematic Review (SR) of 639 qualitative studies was carried out in accordance with the Preferred Reporting Items for Systematic Review (PRISMA) principles. The search strategy was developed using MeSH terms for a range of electronic databases: CINAHL, Pubmed, EMBASE, Medline, AMED and ASSIA were systematically searched in order to identify a variety of literature relevant to the review topic. Four duplicate studies were removed and a further 630 studies were excluded due to being irrelevant. Five qualitative studies were retained and analysed.

**Results:**

The three resultant findings from the literature appraised were i) Experiences and views of Significant Others' (SO) with adult relatives that have HU ii) Support needs of SO and iii) Health professionals interventions to address SO support needs and improve overall HU care. A clear finding was that SO experience difficulties managing HU and this can impact on the relationships that SO and HU patients have. Support needs of SO highlighted were both educational and psychological in nature, with there being a requirement for additional raised awareness within the wider community.

**Conclusion:**

It is essential that healthcare professionals offer support, such as teaching and support groups. In addition, providing interventions into improving family knowledge of diabetes and support with regard to psychosocial, behavioural and practical support for the person with diabetes. Moreover, improving resources for families to improve diabetes care. However, as the literature was of a qualitative nature, future recommendations would be quantitative research into these suggested nursing implementations to quantitatively assess their usefulness in practice.

## 1 Introduction

The introduction of insulin therapy in 1922 (Banting and Best, 2007) dramatically transformed the prognosis of the previously fatal T1DM (Nathan, 2014). Insulin therapy has been widely used to treat and maintain glycaemic control, and to help reduce both macro-and-microvascular complaints (The Diabetes Control and Complications Trial Research Group DDCT, 1993; NICE, 2016). There has been significant research into insulin-related hypoglycaemia (McCall, 2012; Gold et al., 1995). An example of this was the randomised DCCT, which resolved that patients treated with insulin were more susceptible to severe episodes of hypoglycaemia (Nathan, 2014), as a result of insulins increased susceptibility of neuroglycopenia (Gold et al., 1995).

Hypoglycaemia Unawareness (HU) is a complication of diabetes in which neuroglycopenia occurs before the appearance of autonomic symptoms: (i) sweating, ii) hunger, iii) anxiety and iv) tremors (Martin-Timon et al., 2015; Gerich et al., 1991). Clinically a patient will present with a reduced capability in being able to detect hypoglycaemia (Martin-Timon et al., 2015; Shuttlewood et al., 2015). There are many factors that affect the onset of the diabetes complication HU: i) multiple exposure to hypoglycaemic episodes, ii) individuals with a longer duration of diabetes and iii) insulin therapy (Martin-Timon et al., 2015; White, 2007). Reoccurring exposure to hypoglycaemic episodes also results in HU, however, the exact mechanisms that occur in the body are unknown (Martin-Timon et al., 2015).

Severe hypoglycaemia is much higher, (6-fold), in people who experience HU (Martin-Timon et al., 2015). These severe hypoglycaemic episodes pose significant risks such as seizures, fractures, coma and cardiac arrhythmias (Martin-Timon et al., 2015) and studies have shown severe HU episodes to be linked with an increased association of mortality, both at cardiovascular and vascular levels (Gerstein et al., 2008; Zoungas et al., 2010; Hanefeld et al., 2016).

Factors that influence the effectiveness of self-management of HU are dependent on the feelings, attitudes, knowledge and skills of the diabetes patient (Rintala et al., 2013). Previous studies have suggested that family support improves the adherence to self-management strategies of diabetes (Armour et al., 2005). This highlights the importance of caring for the health and wellbeing of these core carers of HU patients (Jorgenson et al., 2003).

Due to the to the presenting nature of HU it is often difficult for the individual to self-treat, as during an episode of hypoglycaemia the individual is cognitively impaired; meaning the person could present confused and uncharacteristic, or at worst be in a comatose state (Elliot and Heller, 2011). SO play a pivotal role in detecting and treating the HU episode (Armour et al., 2005; Elliot and Heller, 2011). The responsibility of looking after people with a recognised medical condition (HU) has being reported as stressful and has been suggested that family members have even more concerns regarding diabetes than the person with the condition (Jorgenson et al., 2003). Therefore, the management and subsequent treatment of HU must be taken seriously by all health professionals (NICE, 2016; WHO, 2017).

There has been significant research into the development of the condition, especially with regard to insulins-inducing effect on HU (White, 2007; Nathan, 2014; DDCT, 1993). Further research is still required to improve our understanding of the mechanisms that determine HU physiology, in order to enhance therapies and treatments (Martin-Timon et al., 2015). Studies have evaluated clinical methods to detect, diagnose and manage HU (Geddes et al., 2007; Gibson, 2009).

However, research on the clinical management of HU appears to be heavily focused on T1DM. Two independent studies showed that although a smaller percentage of T2DM patients were formally diagnosed with HU, those with HU were increasingly likely to experience severe hypoglycaemia (Schopman et al., 2010). Studies also indicated that 56% (n = 25) (Hay et al., 2003) and 47% (n = 70) (Chico et al., 2003) of T2DM presented with asymptomatic hypoglycaemia during continuous blood glucose analysis, whilst undergoing different treatments. This suggests that more T2DM patients may have HU than originally thought, suggesting future research should not discriminate against smaller population groups (Martin-Timon et al, 2015).

Psychological and psychiatric challenges that diabetes patients and their families face in terms of diabetes as a whole condition, for example, stress, coping ability and managing the condition in a social or family setting have also been examined (Weinger and Lee, 2006; Snoek et al., 2014). In addition, research regarding the psychological impact that hypoglycaemia has, in terms of fear and anxiety surrounding and HU have also been investigated (Snoek et al., 2014; Anderbro et al., 2010). Anderbro et al., highlighted a complex relationship between hypoglycaemic episodes and fear and anxiety, showing that patients adopt strategies to avoid hypoglycaemic episodes (2010).

Much of the research already available has a strong medical focus and explores the clinical interventions healthcare professionals could implement (Martin-Timon et al., 2015; Bakatselos, 2011). This literature is important for the treatment and management of the clinical condition, however Elliot and Rankin (2014) express concerns regarding the requirement to consider the psychological needs of both the patient and SO. Due to the demanding nature of diabetes as a chronic illness, its impact extends beyond the physical well-being of the patient (Weinger and Lee, 2006).

To date there are no published SR on the psychosocial impact HU has on SO which provides a rationale for the importance of this research project. This systematic review aims to evaluate significant others' (SO) experiences of caring for diabetes patients with HU. The three outcomes of this research were to 1) examine the experiences and views of significant others with adult relatives with HU; 2) investigate the support needs of significant others and 3) examine healthcare professional interventions to address significant other's support needs with a view to improve overall HU care.

## 2 Materials & methods

We conducted this Systematic Review (SR) in accordance with the Preferred Reporting Items for Systematic Review (PRISMA) principles. The PRISMA checklist was followed to ensure that the appropriate steps were followed in the methodology process.

### 2.1 Data sources and searches

The search strategy was developed using MeSH terms for a range of electronic databases: CINAHL, Pubmed, EMBASE, Medline, AMED and ASSIA were systematically searched in order to identify a variety of literature relevant to the review topic. The reference lists of the studies were reviewed as well and an additional search on www.googlescholar.com was conducted using the key words in order to identify any grey literature that may have been overlooked in the primary search (Bettany-Saltikov and McSherry, 2016).

### 2.2 Search strategy

Search terms used in literature search generated using PICOS (Table 1) with relevant inclusion and exclusion criteria.

### 2.3 Inclusion/exclusion criteria

**Table 1:** Characteristics of SR question using PICOS.

1-Population	Adult >18 Families* or carers looking after adult relatives with Hypoglycaemia Unawareness (HU) *families not necessarily related by blood or marriage but for the purpose of this study ‘Significant Other’ will be used
2-Intervention	≥18 Adults with HU unawareness (Excluding elderly – over 65)
3-Comparison	Experiences and views and subsequent support needs of significant others of adult relatives that have HU
4-Outcomes	Health professionals interventions to address family support needs and improve overall HU care
5-Study	Qualitative, Phenomenological studies, Grounded theory, Descriptive, Ethnography

### 2.4 Population

The Population included SO looking after diabetes patients (Type 1 and 2) who had experienced HU. Due to the remit of this study SO below the age of eighteen were excluded due to the different and complex psychological support ‘young carers’ require.

### 2.5 Intervention

The intervention chosen was adult diabetes patients (≥18) that have experienced HU. Children (≤18) were excluded from the study. Studies including primarily elderly diabetes patients (≥65) were excluded also, as studies found that other factors contribute to HU. For instance, elderly adults present signs of confusion whereas children have been known to cry continuously (Snoek et al., 2014).

### 2.6 Comparison

The comparison was the experiences, views and the subsequent support needs of SO when living with HU patients compared to people not living with this health condition.

### 2.7 Outcomes

The outcome was healthcare professionals' interventions that address family support needs and improve overall HU care.

### 2.8 Study setting

Study designs included in the literature search were both qualitative and mixed-methods articles. The rationale behind selecting both qualitative literature and mixed-methods was due to the demands of the research question.

### 2.9 Study selection

Once the databases were searched potentially relevant articles were reviewed by reading their full titles and abstracts. If the abstract was unavailable or irrelevant then the study was discarded. The selection criteria involved numbering each of the papers placing them into a table with a key to save confusion. After this process four ‘Yes papers’ and five ‘Unsure papers’ remained. The next step required reading the full texts of the Unsure papers in order to decide whether to include them or not. Keeping the same research paper selection form the ‘Unsure papers’ were read in full to make a final decision to include or exclude. Once a decision was made whether to include or exclude the ‘Unsure papers’, the process of selecting papers was finalised. This left five remaining papers.

### 2.10 Critical appraisal

The Critical Appraisal Skills Programme (CASP) tool was used to critically evaluate the four qualitative papers. The Mixed-Methods Appraisal Tool (MMAT) was used for the mixed-methods Paper five.

### 2.11 Data extraction

The process involved going back to the primary articles and highlighting themes relevant to the PICOS framework and using a standardised data collection form (Bettany-Saltikov and McSherry, 2016). The data extraction process was completed by two reviewers (EH and GGA). The studies were independently analysed by EH and GGA. Where there were inconsistencies in data analysis. Any inconsistencies in data analysis were resolved by discussion with a third person.

### 2.12 Data synthesis

The method of data extraction for qualitative data was carried out using Burnard's fourteen stage analysis, which is based on a grounded and content analysis approach (Burnard's Fourteen Stage Analysis, 1991). Grounded theory is a systematic research methodology, which operates inductively and involves the construction of theory through methodic gathering and analysis of data.

As researchers review the data collected, concepts or elements become noticeable, and are identified with codes, which have been extracted from the data. The accumulation of additional data promotes review and re-review and codes can be grouped into concepts, and then into categories. These categories may become the basis for new theory. Thus Grounded theory is the systematic generation of theory from systematic research.

## 3 Results

**Fig. 1:**
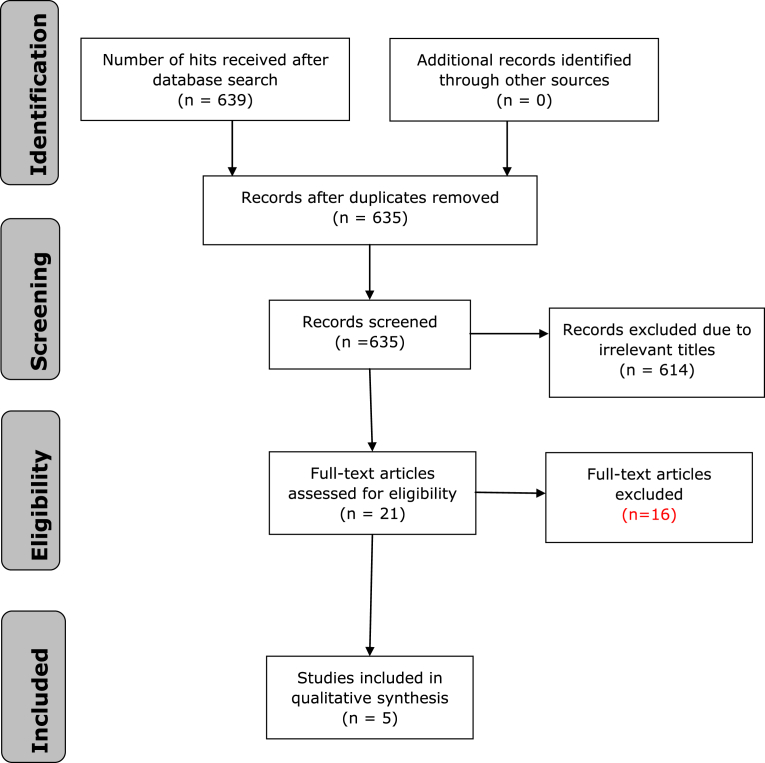
Flowchart of the study selection process for systematic reviews.

After searching the electronic databases using search terms, 639 papers were found, (635 once duplicates were removed). The titles of these papers were examined, leaving 21 papers remaining. Titles and abstracts of these 21 papers were read against the inclusion/exclusion criteria. 5 papers (Table 2) were required to be read in full as it was unclear from their titles and abstracts alone whether they were relevant. As a result of the comprehensive search five papers met the inclusion criteria (Fig. 1). Three main emergent themes were obtained as a result of Significant Others caring for Diabetes Patient's with HU: Theme One: Experiences and views of SO with adult relatives that have HU; Theme Two: Support needs of SO and Theme Three: Health professionals interventions to address SO support needs and improve overall HU care.

### 3.1 Included studies

**Table 2:** Studies included based on PICOS in order to gain understanding of their content.

Study	Title	Participants	Phenomena of interest	Study Method	Study Results
1 Stuckey, H., Mullan-Jensen,C., Kalra, S., Reading, J., Wens, J., Vallis, M., Kokoszka, A., Malek,R., Kovacs Burns, K., Piana, N., Skovlund,S. and Peyrot, M. (2016)	Living with an adult who has diabetes: Qualitative insights from the second Diabetes Attitudes, Wishes and Needs (DAWN2) study	2057 adult family members Mean age: 46 years Study carried out over 17 countries: Algeria, Canada, China, Denmark, France, Germany, India, Italy, Japan, Mexico, The Netherlands, Poland, Russia, Spain, Turkey, United Kingdom (UK) and United States of America (USA). Minimum of 120 participants per country	Lived experiences of those living with an adult who has diabetes	Qualitative: Phenomenological approach	SO wanted to help the with diabetes management. Hypoglycaemia is a concern for SO and diabetes can negatively impact relationships, which can have an emotional impact. SO would like more support in managing diabetes.
2 Lawton, J., Rankin, D., Elliot, J., Heller, S., Rogers, H., De Zoysa, N. and Amiel, S. (2014)	Experiences, Views and Support Needs of Family Members of People with Hypoglycemia Unawareness: Interview Study	Family members of were recruited from type 1 diabetes patients that were participating in the DAFNE-HART study for people with HU, this study was carried out in two diabetes centres within the UK. 24 adults opted in to the study- comprising of 18 partners, 3 parents and 3 adult children. Mean age 45.9 Patients selected from two secondary care diabetes centres in the UK	Significant others that helped diabetes patients in their management of HU	Qualitative- Grounded theory	SO supporting HU patients are in urgent need of emotional support. There is a a requirement for healthcare professional to have a heightened awareness about the condition and more there is a requirement for the development of proactive support for family.
3 King, J., Overland, J., Fisher, M. and White, K. (2015)	Severe Hypoglycaemia and the Role of the Significant Other, Expert, Sentry, and Protector	Seven significant others participated Mean age: 47 Participants that were living in within the Sydney metropolitan and Central Coast regions of New South Wales, Australia.	Participants that had played a pivotal assistive role in the management of severe hypoglycaemia episodes.	Qualitative: narrative inquiry	HU episodes were traumatic and had a heavy impact on the SO lives. SO were heavily relied upon to detect and treat episodes of severe hypoglycaemia. The SO as a result became the expert in the condition, the sentry by being prepared for episodes and the protector by being there to care for the person with HU.
4 Rankin, D., Elliot, J., Heller, S., Amiel, S., Rogers, H., DeZoysa, N. and J Lawton. (2014)	Experiences of hypoglycaemia unawareness amongst people with Type 1 diabetes: A qualitative investigation.	People with Type 1 diabetes who have hypoglycaemia unawareness that required second party assistance from a significant other 38 participants Mean age: 50.6 Participants were recruited from 2 diabetes centres (they were participating in a broader study concerned with HU)	To explore the effects that HU has on everyday life for both individuals with type 1 diabetes and their families involved in their care.	Qualitative: Grounded theory	Since the HU diagnosis reports suggested of a decline in previous pastimes and hobbies. Concerns were raised about the HU patient being a burden to their SO and health care professionals appeared to focus more on the clinical aspect of the condition opposed to the psychological and emotional aspect of the illness.
5 Kovacs, B., Nicolucci, A., Hol, R,. Willaing, I., Hermanns, S., Kalra,S., Wens, J., Pouwer, F., Skovlund, S. and Peyrot, M. (2013)	Diabetes Attitudes, Wishes and Needs second study (DAWN2 tm): Cross-national benchmarking indicators for family members living with people with diabetes.	2057 adult family members Mean age: 46 years Study carried out over 17 countries: Algeria, Canada, China, Denmark, France, Germany, India, Italy, Japan, Mexico, The Netherlands, Poland, Russia, Spain, Turkey, United Kingdom (UK) and United States of America (USA). Minimum of 120 participants per country	To explore the lived experiences of those living with an adult who has diabetes	Qualitative: Mixed Methods Phenomenological approach	Some SO (35.3%) reported that looking after a person with diabetes was burdensome and felt that their emotional wellbeing was impacted. 61.3% of SO worried about complications of diabetes such as HU. SO wanted to be more involved in the care of the person with diabetes but expressed concerns that they did not know enough about the condition.

### 3.2 Methodological quality of included studies

The quality assessment of the papers was carried out using the CASP Tool (CASP, 2017) and MMAT framework. Tables 3 and 4 below illustrate the overall quality of the papers.

#### 3.2.1 CASP qualitative appraisal

**Table 3:** Results of the CASP qualitative appraisal - CASP (2017) About Us [online]. Available at: http://www.casp-uk.net/aboutus.

	CASP Question Number	1	2	3	4	5	6	7	8	9	10	Yes: No: Can't Tell	Judgement of quality (Good/Average/Poor)
1	Stuckey et al. (2016)	✓	✓	✓	✓	✓	✗	✓	✓	✓	✓	9:1:0	Good
2	Latwon et al. (2014)	✓	✓	✓	✓	✓	?	✓	✓	✓	✓	9:0:1	Good
3	King et al. (2015)	✓	✓	✓	✓	✓	✓	✓	✓	✓	✓	10:0:0	Good
4	Rankin et al. (2014)	✓	✓	✓	✓	✓	✓	✓	✓	✓	✓	10:0:0	Good

#### 3.2.2 MMAT criteria appraisal

**Table 4:** Results of the qualitative Mixed Methodology Appraisal Tool (MMAT).

	MMAT Question Number	1	2	3	4	5	6	Yes: No: Can't Tell	Judgement of quality (Good/Average/Poor)
5	Kovacs et al. (2013)	✓	✓	✓	✓	✓	✗	5:1:0	Good
Overall Quality = Number of criteria met/Number of criteria				0.83					

### 3.3 Theme One: experiences and views of SO with adult relatives that have HU

SO have a desire to help and understand HU

Four out of the five studies included in this SR indicated that SO others have a desire to understand HU better and perform a more active role in its management.

*My challenge is remembering how to deal with diabetes as though I had it myself so that I best understand it and can assist the person I live with*

(Stuckey et al. (2016) page 272, col 2, lines 33–35)

Although SO had this strong desire to help, it was identified that they did not know how to help, for instance some SO felt ill prepared in administering glucagon.

*Some (n = 6) being too frightened to administer them (glucagon injections)*

(Lawton et al. (2014) page 113, col 1, lines 43–45)

Certainly in the initial stages of the patient having HU, SO reported feelings of panic and unpreparedness. There was a reoccurring theme in which SO had to adapt and learn how to manage HU.

*Felt inadequate, unprepared or a degree of panic during their first encounter of severe hypoglycaemia*

(King et al. (2015) page 702, col 1, lines 38–49)

HU affects relationship between SO and HU patient

All five papers showed some indication that HU influenced relationships in a negative manner.

*It affected the dynamics of their relationship with their partner/relative/family members*

(Lawton et al. (2014) page 112, col 2, lines 53–55)

There was a sense that HU placed added pressures on the SO, as they were the identified ‘experts’ at detecting the warning signs of HU.

*Shouldered the burden of responsibility of dealing with the severe hypoglycaemia episodes and the issues that ensued*

(King et al. (2015) page 703, col 2, lines 30–32)

For this reason the SO restricted their own lifestyles such as activities, holidays, employment and own health needs.

*The fear of hypoglycaemia removes the freedom to improvise during social activities such as travel, dining celebrations and holidays. Family members do not feel at ease with the person with diabetes in a foreign country when there is a risk of needing a hospital or urgent treatment. Diabetes removes the freedom to be spontaneous*

(Stuckey et al. (2016) page 273, col 2, lines 12–17)

Through these restrictions feelings of resentment emerged and SO admitted to sometimes feeling burdened by HU. These negative feelings towards HU placed an emotional strain on the SO- HU patient relationship. Findings are suggestive of power imbalances in relationships with the SO taking on a paternalistic role.

*I don't want to be too much of a babysitter, as it emotionally gives an imbalance in our relationship*

(Stuckey et al. (2016) page 275, col 1, lines 42–43)

#### 3.3.1 Emotional wellbeing of SO affected

All five papers discuss to some extent how the emotional wellbeing of the SO is affected by HU. This was highlighted by the numerous traumatic episodes that SO were witness to.

*Severe hypoglycaemia episodes was a distressing experience for the significant others*

(King et al. (2015) page 700, col 2, lines 29–30)

Following these harrowing accounts SO had concerns regarding leaving their relatives unsupervised.

*Her husband had a hypoglycaemic episode while driving that had resulted in her being “constantly on alert, frightened, and apprehensive*

(Lawton et al. (2014) page 111, col 3, lines 52–54)

Another factor that caused emotional stress and anxiety was the unpredictable and uncharacteristic behaviour that HU patients presented during episodes.

*Several participants were met with verbally abusive behaviour by their spouses on some occasions.*

(King et al. (2015) page 702, col 1, lines 10–11)

Certain cases described episodes that displayed violence and aggression, in which SO admitted to fearing for their own personal safety.

*In extreme cases, participants reported physically ‘fighting’ (R13) others off*

(Rankin et al. (2014) page 186, col 2, lines 43–45)

This build-up of emotions resulted in the SO feeling resentful of their relative, which they subsequently felt guilty about. This emotional turmoil experienced by SO was heightened by poor and interrupted sleep patterns due to concerns regarding nocturnal hypoglycaemia.

*Very poor, interrupted sleep owing to their worries that the person with HU would slip into a coma or exhibit violent behaviour toward them during the night*

(Lawton et al. (2014) page 112, col 1, lines 53–56)

### 3.4 Theme Two: support needs of SO

Significant others need more educational support

Findings were suggestive of SO lack of education about HU particularly in the initial stages of diagnosis.

*Upon diagnosis, many family members initially knew little about the disease and the imposing wide-ranging changes that could occur in the lives of both the person with diabetes and the family member*

(Stuckey et al. (2016) page 273, col 1, lines 28–31)

This lack of knowledge regarding HU left SO feeling ill-equipped both physically and psychologically unprepared for episodes of HU.

*They recall dramatic episodes linked to hypo- glycaemia and their inability to know what to do, how to react, or to understand what is happening*

(Stuckey et al. (2016) page 273, col 1, lines 37–39)

#### 3.4.1 Psychological support required for SO

All of the papers in the SR spoke about the emotional consequences HU had on SO.

*Hard to live with” and ‘‘unbearable,” but family members recognised that often the person is not aware he/she is ‘‘Moody.” Some family members deal with this by suppressing their feelings about the consequences in mood*

(Stuckey et al. (2016) page 275, col 1, lines 30–33)

Two of the papers explored this topic in further depth and reported of consequences such a burn out as a result of caring for HU relatives.

*Extreme burnout and fatigue was also apparent*

(Lawton et al. (2014) page 112, col 1, lines 27)

It was apparent that SO had not spent time discussing their thoughts and feelings related to caring for a person with HU before and some felt the process of interviewing for the research process cathartic.

*Few significant others had told their stories before, and the effect was akin to sharing the burden*

(King et al. (2015) page 704, col 1, lines 46–48)

Increased public awareness about HU

The research suggested a lack of public awareness with regard to HU as condition, this was a concern for SO as they feared the potential judgement the person with HU could be subject to.

*Community or societal discrimination against the person with diabetes was found to exist*

(Kovacs et al. (2013) page 784, col 1–2, lines10-11/1)

This lack of education to the wider public was also a concern in respect to the level of societal support and understanding available to HU as a condition.

*Did not want his “odd” behaviour during a severe hypoglycaemia episode to compromise his “credibility, both personally and professionally, and his dignity.”*

(King et al. (2015) page 703, col 1, lines 36–38)

#### 3.4.2 Safeguarding SO

The requirement for safeguarding was highlighted within the papers researched. Although every patient with HU presents uniquely, the research reports of incidences in which HU patients have displayed violence and aggression to their SO.

*They exhibited hostile, aggressive and sometimes very violent behaviour*

(Rankin et al. (2014) page 188, col 2, lines 42–44)

Reports of this aggressive behaviour has left SO feeling vulnerable and afraid of their relative.

*Being very fearful for their own safety*

(Rankin et al. (2014) page 188, col 2, lines 45–46)

Arguably, the HU patient is unaware of their actions during a HU episode but regardless of this a zero tolerance strategy should be applied when it comes to the safety and wellbeing of SO involved.

*Can present aggressive and sometimes violent behaviour and that this is not a reflection of the person's personality (paper 3)*

(King et al. (2015) page 704, col 2, lines 31–33)

### 3.5 Theme three: health professionals interventions to address SO support needs and improve overall HU care

Healthcare professionals offer educational support to SO about HU

All five of the research papers mentioned the requirement for an increased level of educational support for SO, due to the appreciation that they are a big role in the patient's life.

*‘Support is needed for family members, because they are one of the greatest resources to provide exercise or dietary support to the person with diabetes’*

(Stuckey et al. (2016) page 275, col 2, lines 36–38)

The role of the health professionals could arguably be pivotal in supplying this education to SO. SO were particularly keen for information regarding what to expect from HU.

*If someone could just put a couple of lines in to say that these things may happen!*

(Rankin et al. (2014) page 187, col 2, lines 33–34)

Specific education goals expressed by SO were: nutritional knowledge, awareness of diabetes complications and administering emergency medicines such as glucagon.

*Family members requested more knowledge and more awareness about complications and diabetes' e.g. ‘nutritional knowledge*

(Stuckey et al. (2016) page 276, col 1, lines 6–8)

*Ensure that caregivers are offered education and information about applying hypoglycaemia management. Health professionals could also consider extending similar kinds of support to family members of people with type 2 diabetes using insulin or sulfonylureas*

(Lawton et al. (2014) page 114, col 1, lines 41–47)

#### 3.5.1 Support groups for SO

The research investigated highlighted the request of some sort of support network for SO.

*Support group programs, better community resources, and family- inclusive support from clinicians’*

(Stuckey et al. (2016) page 275, col 2, lines 48–50)

SO expressed their need for the groups to contain people in similar situations in order to gain tips and solutions to real life situation that they can relate to.

*Reassurance, feedback, and emotional support from people in the same situation as themselves to help overcome their feelings of isolation, resentment, and sometimes guilt*

(Lawton et al. (2014) page 113, col 2, lines 14–18)

These support groups could be in the form of online forums, websites or groups in clinics.

*Dedicated support groups could be set up within hospital diabetes clinics or other settings*

(Lawton et al. (2014) page 114, col 1, lines 3–6)

#### 3.5.2 SO invited to diabetes appointments

The evidence gathered reported of the benefit of having SO attending clinical appointments with the HU patient. These consultations would provide opportunity for healthcare professionals to opportunistically breach the subject of coping with the SO.

*We would also encourage health professionals, such as family members' own general practitioners, to ask, opportunistically, how they are coping during consultations and routine health checks*

(Lawton et al. (2014) page 114, col 1, lines 24–29)

By inviting SO to diabetes consultations would increase the chances of effective relationships being built between healthcare professionals and carers to opportunistically improve patient care.

*Building an effective relationship between the diabetes health care team and the person with diabetes and his or her family*

(King et al. (2015) page 704, col 2, lines 3–5)

As HU patients cannot always coherently recall an episode of HU, reliance on the SO account is necessary. Having a more accurate presentation of the HU episode will potentially be beneficial in the diagnosis and treatment of the condition.

*Worried that, due to poor recollection, the person with HU might be underreporting episodes of severe hypoglycaemia*

(Lawton et al. (2014) page 113, col 2, lines 8–12)

#### 3.5.3 Healthcare initiatives into preventing HU in the future

The research gathered suggested improvement to be implemented to help reduce HU's severity and future development.

*Hoping for better healthcare in general with primary goals of diabetes prevention so the disease would not happen to others*

(Stuckey et al. (2016) page 276, col 1, lines 17–19)

A general resounding theme suggested that healthcare professionals offer educational and psychological support, with a particular focus on tight glycaemic control.

*Hypoglycaemia unawareness can be reversed through strict avoidance of hypoglycaemia, 14–16,31 albeit, extensive professional input may be required*

(Rankin et al. (2014) page 189, col 1, lines 15–19)

Clinical research into HU and its prevention

An emergent theme from the data analysed was for further clinical research to provide beneficial treatment and prevention of HU in the future.

*That clinical effort should continue to be directed toward diagnosing HU and offering effective interventions to help patients restore awareness of hypoglycaemia*

(Lawton et al. (2014) page 113, col 3, lines 42–46)

## 4 Discussion

Hypoglycemia unawareness (HU) is defined at the beginning of neuroglycopenia prior to the appearance of autonomic warning symptoms. It is a major limitation to achieving tight glycaemic control and improved quality of life. HU occurs in approximately 40% of people with type 1 diabetes mellitus (T1DM) and with less frequency in T2DM. This systematic review aimed to appraise significant others' (SO) experiences of caring for diabetes patients with HU. GGAlthough three main findings were obtained from this qualitative systematic review, a strong finding was that Significant Others (SO) experience problems when attempting to manage hypoglycaemic unawareness (HU) in patient with diabetes, which impacts on the relationships between SO and HU patients.

### 4.1 Theme One

The experiences and views of SO caring for diabetes patients that have HU

The three subthemes of this main theme were: i) SO's have a desire to help and to understand HU ii) HU affects the relationship between SO and HU patient and iii) Emotional wellbeing of SO is affected. SO expressed a desire to help the patient with HU (Stuckey et al., 2016), however were concerned that they did not feel competent in doing so (Lawton et al., 2014). This feeling of incompetence was acquired through witnessing traumatic HU episodes in which SO felt unsure of how to act (King et al., 2015). Following this SO felt they needed to be more vigilant and prepared for future occurrences to prevent similar traumatic experiences happening again (King et al., 2015). Similar findings have been found in carers of people who have had strokes. Gillespie and Campbell state that the effects of stroke extend beyond the patient (2011). In stroke survivors, the risk of a reoccurrence is fatally high (Burn et al., 1994), often this means that victims of strokes and their SO feel as though they must be prepared. This risk of reoccurrence of deterioration of health accompanied with the pressure to be prepared for these episodes in stroke patients can be compared to HU patients.

Research suggests that carers of people with long-term illnesses become experts by experience in the condition (CQC, 2018; Burn et al., 1994; King et al., 2015). Findings showed greater relationship satisfaction when the SO was more knowledgeable of the patient's condition as they were able to understand the patient's needs better (Gillespie and Campbell, 2011; Williams, 1993). A study by Haley et al. (2009), aimed to quantify the benefits SO (n = 75) experienced when caring for a person post-stroke. Results found that 67% of people believed they acquired new skills and 81% felt a sense of self-satisfaction though caring for family members (Haley et al., 2009).

It can be concluded that SO desire to understand HU stems from: i) having the ability to know how to act in a medical emergency to avoid traumatic occurrences and ii) enhancing their own self-satisfaction through caring (Haley et al., 2009).

In terms of how HU affects the relationships between SO and HU patients, the studies findings suggested that the relationship between the SO and the patient was affected negatively by the condition HU (Lawton et al., 2014). Reports indicated SO restricted their lifestyles to care for the HU patient, which provoked feelings of resentment and annoyance (King et al., 2015; Stuckey et al., 2016). Olson (2015) has explored the connotations of the term ‘carer’ and the effect on relationships. Caring responsibilities are often assumed by SO (Given et al., 2012) but this can add extra pressure and in some cases feel burdensome (Olson, 2015; Gallicchio et al., 2002). Olson explains that if an illness takes precedence within a relationship, it extinguishes the capacity for meaningful interaction in a relationship (2015). As a result of illness, the SO becomes a carer first rather than a mother, father, spouse etc. This identity change within a relationship can be upsetting and causes a period of mourning for the ‘social death’ of their family member affected by illness (Olson, 2015). This role change has been observed in other chronic illnesses such as cancer, dementia and stroke (Olson, 2015; Given et al., 2012; Gallicchio et al., 2002). Findings suggested that the impact chronic illness places on carers was substantial enough to cause depression in individuals (Gallicchio et al., 2002), and that as caregivers were the primary sources of support during chronic illness, adequate healthcare-support is required for them (Given et al., 2012).

On top of added caring duties, SO felt HU removed the ability to be spontaneous (Stuckey et al., 2016). Rintala et al. (2013) published similar findings in a SR they conducted into interrelations between diabetes patients and their families (including 29 studies, n = 36000). Findings revealed barriers to being impulsive, such as dietary requirements, managing hypoglycaemia and overprotective behaviour (Rintala et al., 2013).

All five studies discussed the emotional wellbeing of the SO being affected by HU. Research by Gillespie and Campbell confirms that increased stress on relationships caused by illness can decrease relationship satisfaction and emotional wellbeing (2011). Rintala et al. (2013) express how diabetes affects not only the patient but SO also; stating that they experience more concerns about risks associated with the condition than the patient themselves. A qualitative interview study explored how chronic illness affects the quality of life of caregivers (n = 49), over 50% of participants reported that their emotional wellbeing was in some way affected by the burden of caregiving responsibilities (Wittenberg et al., 2013).

The impact that caring responsibilities have on a carer's emotional wellbeing is not a new phenomenon, with Fengler and Goodrich (1979) defining carers as ‘hidden patients’. Bell et al. (2001) explained that caregiving responsibilities have psychological aspects (anxiety, guilt and worry) as well as physical consequences of care delivery (tiredness, reduced social life and employment restraints).

Reasoning behind SO reduced emotional wellbeing has been associated with added challenges SO face including: i) physical and technical encounters that involve learning about health conditions ii) emotional strains, whereby carers mourn the social death of their family member iii) social demands, as care responsibilities remove the ability to be spontaneous and iv) psychological stress, with carers reporting higher levels of anxiety and depression due to stress (Given et al., 2012). As a result of these psychological and physical consequences emotional wellbeing is affected (Bell et al., 2001; McConaghy and Caltabiano, 2005).

### 4.2 Theme Two: support needs of SO

In accordance with the Care Act, healthcare professionals and other healthcare professionals are under a legal obligation to identify and meet these carer support needs (2014). The following support needs were found within the literature explored: ii) SO need more educational support ii) Psychological support required for SO iii) Increased public awareness about HU and iv) Safeguarding SO. Bonsignore (2018) states that diabetes education is imperative, as there are many factors that contribute to management, such as: i) nutrition, ii) physical activity, iii) medicines management, iv) monitoring blood sugar v) and psychosocial adjustments. Due to the substantial role SO have in the management of HU (Armour et al., 2005; Elliot and Heller, 2011), it is important that SO have a good knowledge of HU to improve overall evidence-based care.

Diabetes patients expressed their appreciation for being able to share and confer about diabetes related matters with their SO, (Rintala et al., 2013). Van den Heuvel et al. (2000) suggest that caregiving strategies should focus on education and teaching whilst (n = 28) reported an increased sense of control and a reduced sense of anxiety once family members were given more knowledge (1986).

This stance was further resonated in an intervention study (n = 936) (Murphy et al., 1995), whereby more informed carers experienced less negative emotions and health problems (F = 4.5, P < 0.05) than less knowledgeable carers (F = 4.21, P < 0.05). It is important that SO are equipped with the knowledge regarding HU in order to offer informed advice when required and to feel in control of the care they deliver.

#### 4.2.1 Psychological support required for SO

The psychological implications SO experience as a result of HU need addressing (Lawton et al., 2014; Stuckey et al., 2016; Bonsignore, 2018; Corden and Hirst, 2011). However, in order for SO to access the appropriate psychological support services they need to accept the title of ‘carer’ and ask for support, which may present certain barriers (Corden and Hirst, 2011).

#### 4.2.2 Increased public awareness about HU

Research spoke of negative experiences towards HU patients and their SO from wider society (Kovacs et al., 2013). SO and HU patients were subject to discriminative behaviours and SO worried especially about HU episodes causing ‘embarrassment’ in public (King et al., 2015). Browne et al. (2014) state that there is substantial research into stigma related illness such as HIV/AIDS and obesity but highlight gaps in literature for the stigma associated with diabetes; research relating to HU specific stigma is therefore limited. Health stigma defines the experiences of exclusion, rejection and stereotyping witnessed by individuals with an illness, due to preconceived social ideas (Link, and Phelan, 2001; Weiss et al., 2006). Browne et al. (2014) found that the impact of diabetes stigma was detrimental to both the patient and their SO; this stigma affected their emotional wellbeing, identity, ability to disclose details about the condition, and social relationships.

The International Diabetes Federation (IDF) (2013) has identified diabetes-related stigma as problematic and encourages urgent attention to this matter. An interview study conducted by Irani et al. (2014) explored strategies to overcome diabetes related stigma (n = 74). Irani et al. (2014) recommend education on a societal basis, to remove false assumptions and reduce stigma. These findings may not be applicable to different cultures, due to the Iranian study setting in which this study was conducted.

#### 4.2.3 Safeguarding SO

The research investigated reported incidents in which abusive or aggressive behaviour was displayed by HU patients towards the SO (King et al., 2015; Rankin et al., 2014). One paper reported SO being fearful for their own safety (Rankin et al., 2014). As highlighted in previous literature, every HU episode manifests itself in various ways (King et al., 2015; Rankin et al., 2014), for this reason every individual would have to be considered uniquely. However, The Care Act (SCIE, 2014) provides a legal framework that aims to protect adults from abuse. Therefore if cause for concern arose, it would be within the healthcare professionals legal duty of care to follow the correct policy for safeguarding (NMC, 2015).

### 4.3 Theme Three: health professionals' interventions to address SO support needs and improve overall HU care

Interventions highlighted were: i) Healthcare professionals offer educational support to SO about HU ii) Support groups for SO and iii) SO invited to diabetes appointments.

#### 4.3.1 Healthcare professionals offer educational support to SO about HU

The NMC Code states that healthcare professionals must explain health conditions and ongoing treatment, in a way patient and families understand (NMC, 2015). Tamura-Lis explains that effective health education significantly impacts on patient safety and the quality of care they receive (2014). Results from ‘Rethink’, a national carer survey, showed that carers reported fewer complaints about their daily lives when they received adequate information (Pinfold and Corry, 2003). It is important that carers have a good understanding of their health conditions, in order to receive treatment that best suits their needs and to adopt health benefitting behaviours (Bollard and Hill, 2016). According to Bollard and Hill (2016) patient and families often experience an inability to understand information regarding their health but this is somewhat overlooked by the health care team. Ultimately, the healthcare professional team, of which healthcare professionals are part of, are at fault if there is a failure in patient-family understanding (Bollard and Hill, 2016).

In order to ensure patients and families have a clear understanding of the health literature, certain strategies can be utilised by healthcare professionals. Communication is integral to nursing practice, so much so that the Chief Nurse of England included Communication within the 6 C's of nursing care (Cummings, 2012). Clear communication methods should be used both in written and verbal form; this should include the use of everyday language and avoiding medical jargon (NMC, 2015; Brega et al., 2015).

The teach-back method is used to ensure that the information taught by the nurse is retained and understood (Brega et al., 2015; Dinh et al., 2016). The method has been appraised as one of the most successful and evidence-based methods in patient and family education (Tamura-Lis, 2014). The Teach-Back Method involves the nurse explaining the healthcare literature; patient-family understanding is verified when they are able to reiterate back the information in their own words (Tamura-Lis, 2014). The teach-back method along with effective communication could be implemented in future practice in order for healthcare professionals to teach SO about HU.

#### 4.3.2 Support groups for SO

Themes from the literature were that SO would like added support when caring for HU patients, in the form of family-inclusive support or group support from people in similar situations (Stuckey et al., 2016). The literature search found no literature regarding support groups for SO when caring for diabetes patients. Diabetes UK (2017) do offer support groups for people who have diabetes but there was no mention on their website about support for SO. The rationale behind group support groups was to gain a sense of reassurance and to overcome feelings of isolation (Lawton et al., 2014).

This human desire not to be isolated within a circumstance can be explained by Baumesiter and Leary's theory of belongingness (1995) which suggests that humans have developed a desire for closeness and social belonging as this gives us a sense of security (Baumeister and Leary, 1995). On the other hand, when ostracised, humans can experience psychological distress (Sommer et al., 2001). Baumesiter and Leary's (1995) theory is further reinforced by Maslow's Hierarchy of needs, (McLeod, 2017). The five stages of Maslow's model are divided into needs that humans strive to meet. The lower level needs (bottom of the pyramid), need to be met first in order to progress up the pyramid to higher level needs (McLeod, 2017). According to Maslow (1968), self-actualisation is defined as the person's ability to recognise their potential, but this process is continual as it is not possible to reach a static state of self-actualisation. Interestingly, reasonably low down in Maslow's hierarchy is ‘Love and Belonging’, suggesting the requirement for social support is relatively high (McLeod, 2017).

A study evaluating the impact that support groups had on caregivers of stroke patients (n = 240) showed positive results (Malini, 2015). Following attendance to support groups, it was found that there was a positive increase in the strength of family systems (Malini, 2015), these findings are consistent with another study by Van Den Heuvel et al. (2000). Van den Heuvel et al.'s (2000) findings suggest support groups are effective nursing strategies that can be implemented to improve overall well-being for SO.

#### 4.3.3 SO invited to diabetes appointments

Findings from the literature indicated the benefits of inviting SO to diabetes clinical appointments (King et al., 2015), giving the health professionals availability to opportunistically ask the SO how they are coping, as well as finding out potentially important information about the patient (Lawton et al., 2014).

It is well documented within the nursing literature that involvement of carers is crucial to quality patient care (The Kings Fund, 2014; Cross-Government Publication, 2010; Francis, 2010). In some circumstances it may be relevant for the carer and the clinician to carry out an assessment of the patients' needs together (The Princess Royal Trust, 2009) as SO may be able to help provide healthcare professionals with information about the patient (The Kings Fund, 2014). During HU episodes the patient is cognitively impaired and as a result their recall of the event is limited. Due to this reduced ability to recall events, the diagnosis and subsequent treatments given by healthcare professionals may be compromised, however having the SO present during consultations could alleviate this barrier to communication.

The Triangle of Care is a model of care that brings together patients, their SO and health professionals (Carers Trust, 2013; Worthington et al., 2014). Through six key standards, the model's holistic approach aims to ultimately improve collaborative, person-centred patient care (Carers Trust, 2013; Worthington et al., 2014; RCN, 2017). Applying models such as the triangle of care (Carers Trust, 2013; Worthington et al., 2014), can bring benefits to SO as well as the patient as they can learn new skills and experience enhanced self-confidence (Orr et al., 2013).

It is important to note that involvement of SO is dependent on the agreement of the individual they are caring for, healthcare professionals should refer to policy and guidelines regarding issues of consent and discussion of the ‘carer role’ (NMC, 2015; Slade et al., 2007).

### 4.4 Future research

Future clinical research into HU and its subsequent prevention was also a key finding (Lawton et al., 2014). Martin-Timon et al. (2015), states that research has begun to uncover some of the underlying mechanisms in which the central nervous system responds to hypoglycaemia (Bakatselos, 2011). By understanding of these mechanisms, treatment can thus be improved, however until this is fully achieved it is important healthcare professionals highlight risks and offer hypoglycaemia prevention strategies to patients.

Quantitative research should be undertaken into the effectiveness of healthcare interventions such as i) support groups, ii) education and iii) inviting SO to diabetes appointments and the physiological causes of everyday insulin and medication might have on HU patients.

## 5 Conclusion

In terms of patient support, it is essential that healthcare professionals offer support, such as teaching and support groups. In addition, providing interventions into improving family knowledge of diabetes and support with regard to psychosocial, behavioural and practical support for the person with diabetes. Moreover, improving resources for families to improve diabetes care. However, as the literature was of a qualitative nature, future recommendations would be quantitative research into these suggested nursing implementations to quantitatively assess their usefulness in practice.

## Declarations

### Author contribution statement

E. Hartill, G.G Adams: Conceived and designed the experiments; Performed the experiments; Analyzed and interpreted the data; Contributed reagents, materials, analysis tools or data; Wrote the paper.

R. B Gillis, S. Imran Jiwani, N. Recchia, A. Meal: Analyzed and interpreted the data; Contributed reagents, materials, analysis tools or data; Wrote the paper.

### Funding statement

This work was supported by the InDependent Diabetes Trust.

### Competing interest statement

The authors declare no conflict of interest.

### Additional information

No additional information is available for this paper.
